# Pre-transplant depression as a predictor of adherence and morbidities after orthotopic heart transplantation

**DOI:** 10.1186/s13019-017-0626-0

**Published:** 2017-07-25

**Authors:** Maja Delibasic, Burhan Mohamedali, Nikola Dobrilovic, Jaishankar Raman

**Affiliations:** 1 Department of Internal Medicine, Mercy Hospital, Chicago, IL USA; 2 Departments of Internal Medicine, Rush University Medical Center, Il, Chicago, 60612 USA; 3 Cardiovascular and Thoracic Surgery, Rush University Medical Center, Rush University Medical Center, 1725 W Harrison, Suite 1156 POB, Chicago, IL 60612 USA

**Keywords:** Depression, Heart failure, Orthotropic heart transplantation, Hospitalization, Rejection, Survival

## Abstract

**Background:**

Psychosocial factors are useful predictors of adverse outcomes after solid organ transplantation. Although depression is a known predictor of poor outcomes in patients who undergo orthotopic heart transplantation (OHT) and is actively screened for during pre-transplant evaluation, the effects of early identification of this entity on post-transplant outcomes are not clearly understood. The purpose of this study was to evaluate the impact of pre-transplant depression on outcomes after OHT.

**Method:**

In this retrospective study, 51 patients that underwent psychosocial evaluation performed by a social worker prior to the transplant and followed up in our center post-transplant were enrolled. Patients were stratified by the presence/absence of depression during the initial encounter. Primary end-points were overall survival, 1st-year hospitalizations, overall hospitalizations, rejections, and compliance with medications and outpatient appointments.

**Results:**

Depressed patients were 3.5 times more likely to be non-compliant with medications; RR = 3.5, 95% CI (1.2,10.2), *p* = 0.046 and had higher incidence of first year hospitalizations (4.7 *±* 3.1 vs. 2.2 ± 1.9, *p* = 0.046), shorter time to first hospitalization 25 days (IQR 17–39) vs. 100 days (IQR 37–229), *p* = 0.001. Patients with depression also had higher overall hospitalizations (8.3 *±* 4.4 vs. 4.6 ± 4.2, *p* = 0.025,) and higher number of admissions for infections (2.8 *±* 1.3 vs. 1.5 ± 1.4, *p* = 0.018) compared to patients without depression. There were no statistically significant differences in total number of rejections or compliance with outpatient appointments. Kaplan-Meier survival analysis did not reveal differences between the two groups (mean 3705 vs. 3764 days, log-rank *p* = 0.52).

**Conclusion:**

Depression was a strong predictor of poor medication compliance and higher rates of hospitalization in transplant recipients. No difference in survival between depressed and non-depressed patients after OHT was noted.

## Background

Depression continues to remain a strong predictor of morbidity and mortality in patients with chronic medical conditions [1, 2]. Cardiac transplantation is the gold standard treatment for end-stage heart failure. Patients with a condition so advanced as to require transplantation may experience pre/post-postoperative anxiety and depression [3–5]. The prevalence of major depressive disorder in patients on a heart transplant waiting list is almost 24%. Further increases in the intensity of the symptoms during the waitlisted period have also been reported [6, 7].

Many risk factors for post-cardiac transplant morbidity and mortality are well –defined and include: heart failure etiology, recipient age, donor age, body mass index, history of previous transplantation, impaired renal function, number of rejection episodes, and cardiac allograft vasculopathy [8].

Although psychosocial risk factors are used to determine eligibility for placement on a heart transplant waiting list, few studies have examined the validity of such factors in predicting post-transplant outcomes. Several studies reported no association between psychosocial factors and mortality [9–12], while others have found that several psychiatric risk factors and social/demographic characteristics correlated with post-transplant morbidity [8]. As a result, the role of psychosocial factors on heart transplant outcomes requires further evaluation [13]. In this study we investigated the prognostic utility of “pre-transplant depression” on survival and morbidity after heart transplantation.

## Methods

### Patient data

This study was approved by our Institutional Review Board. It is a retrospective, study, examining 51 consecutive patients who underwent OHT at a single institution between 1999 and 2013. Relevant clinical data was extracted from United Network for Organ Sharing Transplant (UNOS), paper charts, and electronic medical records. Each patient had a full social worker psychosocial evaluation on record as part the preoperative transplantation work-up.

### Social and demographic variables

Demographic variables included age, sex, race, citizenship and distance from the transplant center based on patient United States Postal Service zip code. All recipients underwent standardized evaluation by the heart transplant social worker. Social factors were defined as those variables that were present in the social history section of the assessment for transplant candidacy conducted by the transplant multidisciplinary team. These variables included each candidate’s marital status, employment status, access to phones, type of medical insurance, educational level, transplant readiness and the presence of social support. Any history of mental health problems, such as depression, bipolar disorder, anxiety, substance abuse (tobacco, alcohol and/or recreational drugs) was included. Patients were stratified by the presence or absence of depression during the initial visit using the self-scoring Beck Depression Inventory II. Depressed patients were further evaluated and treated by the psychiatry service. Inpatient and outpatient medical records were reviewed for antidepressant medication compliance.

### Outcomes data

Survival data was obtained from the electronic medical record and UNOS data. Data on first hospitalization, overall hospitalizations, as well as cause of hospitalization (infection, rejection or other), compliance with outpatient follow-up appointments and medications was obtained. Rejection data regarding both acute cellular rejection and antibody mediated rejection were collected from biopsy data and electronic medical records. Hospitalizations for infections, defined as any hospitalization for documented fever, bacteremia, viremia, fungemia and pneumonia, were tabulated.

### Statistical analysis

Pearson correlation coefficients were used on continuous variables. Independent t-tests and chi square-tests of independence were used to compare groups for continuous and discrete dependent variables respectively. A *p*-value of <0.05 was considered significant. Kaplan-Meier survival analysis was performed to distinguish survival in the two groups.

## Results

**Fig. 1:**
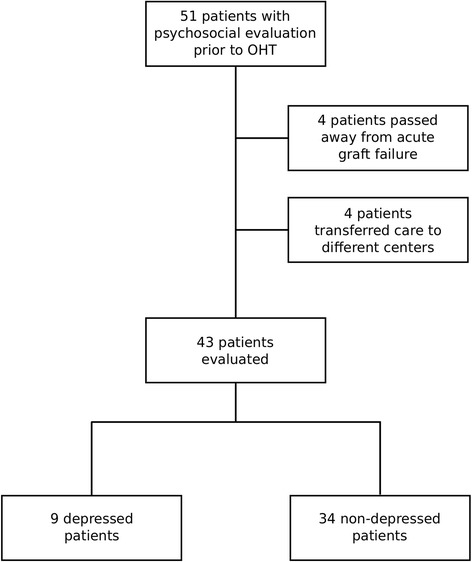
Study Design

Fifty-one patients met inclusion criteria. Four patients who died within the first 30 days from acute graft failure and four other patients who transitioned their care to other centers after transplantation were excluded from the analysis. (Figure 1) of the 43 patients enrolled in our study, 9 patients (20.9%) were diagnosed with depression prior to OHT and 34 patients (79.1%) did not have depression. There was no significant difference in baseline characteristics of the two groups (Table 1). The depressed group had slight predominance of the female sex (44.5% vs. 23.5%, *p* = 0.219). Mean age was similar between two groups (57.5 *±* 12.4 vs. 56.4 *±* 12.3, *p* = 0.816). In both depressed and non-depressed groups, patient were predominantly African American (55.5% vs. 41.2%, *p* = 0.353), unemployed (88.9% vs. 91.2%, *p* = 0.861) and had a history of tobacco abuse (66.7% vs. 70.6%, *p* = 0.822).

**Table 1:** Baseline Demographics and Clinical Data in Depressed and Non-Depressed Group of Patients

	Depressed patients	Non-depressed patients	
	*N* = 9	*N* = 34	*p*
Age (years)	57.5 ± 12.4	56.4 ± 12.3	0.816
Gender			
Male	55.5% (5)	76.5% (26)	0.219
Female	44.5% (4)	23.5% (8)	
Race			
Caucasian	33.3% (3)	35.3% (12)	0.353
African American	55.5% (5)	41.2% (14)	
Hispanic	11.2% (1)	17.6% (6)	
Asian	0	5.9% (2)	
Partner Status			
Partner	55.5% (5)	58.8% (20)	0.861
No Partner	44.5% (4)	41.2% (14)	
Employment Status			
Not Employed	88.9% (8)	91.2% (31)	0.836
Employed	11.1% (1)	8.8% (3)	
Insurance Type			
Government	66.7% (6)	61.7% (21)	0.789
Private	33.3% (3)	38.3% (13)	
Prior Tobacco History			
Yes	66.7% (6)	70.6% (24)	0.822
No	33.3% (3)	29.4% (10)	
Prior Alcohol Abuse			
Yes	33.3% (3)	58.8% (20)	0.178
No	66.7% (6)	41.2% (14)	
Prior Illicit Drug Use			
Yes	11.1% (1)	14.7% (5)	0.784
No	88.9% (8)	85.3% (29)	

There was no difference in baseline characteristics between the two groups

**Fig. 2:**
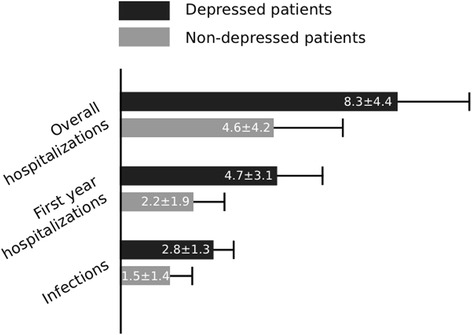
Clinical Outcomes in Transplant Recipients with and without Depression

Depressed patients, compared to the control group, had higher incidence of first year hospitalizations (4.7 *±* 3.1 vs. 2.2 ± 1.9, *p* = 0.046), had higher overall number of hospitalizations during a follow up period (8.3 *±* 4.4 vs. 4.6 ± 4.2, *p* = 0.025), and had a shorter time to first hospitalization post-transplant (25 (IQR17-39) vs. 100 (IQR 37–229), *p* = 0.001). Patients in the depressed cohort had higher number of admissions for infections (2.8 *±* 1.3 vs. 1.5 ± 1.4, *p* = 0.018) (Table 2, Fig. 2). Patients with a pre-transplant diagnosis of depression were 3.5 times more likely to be non-compliant with medications (RR = 3.5, 95% CI (1.2-10.2); *p* = 0.046). There was no difference in incidence of rejections or compliance with outpatient appointments. Kaplan-Meier survival analysis did not reveal any statistically significant difference in survival between the two groups (3705 vs. 3764 days, log rank *p*-value = 0.52)

**Table 2:** Clinical Outcomes in Transplant Recipients with and without Depression

	Depressed patients	Non-depressed patients	
	*N* = 9	*N* = 34	*P* VALUE
Medication Compliance (%)	20.9	79.1	0.046
Time To First Hospitalization(Days)	25(IQR 17–39)	100(IQR 37–229)	0.001
First Year Hospitalizations	4.7 ± 3.1	2.2 ± 1.9	0.046
Overall Hospitalizations	8.3 ± 4.4	4.6 ± 4.2	0.025
Overall Infections	2.8 ± 1.3	1.5 ± 1.4	0.018
Overall Rejections	2.0 ± 1.5	1.2 ± 1.4	0.136
No Show Rate (%)	14 ± 6	12 ± 7	0.398
Overall Survival (Days)	3705	3764	0.52

.

## Discussion

Depression after cardiac surgery is well described [14]. Numerous studies have demonstrated an association between increased morbidity and mortality in depressed patients with concomitant cardiovascular disease compared to non-depressed patients [15–17]. Depression is a known marker of poor outcomes after transplant. The International Society of Heart and Lung Transplantation (ISHLT) recommends routine screening for depressive symptoms [18]. In our study we found that the outcomes between patients with and without pre-transplant depression did not differ in survival, total number of organ rejections, and compliance with outpatient appointments. Depressed patients had higher overall hospitalizations, higher number of first year hospitalizations and shorter time to hospitalization compared to the non-depressed cohort. Furthermore, depressed patients had higher rates of admission for infections and were more likely to be non-compliant with medications.

There is a high incidence of depression after OHT, often requiring regular screening and early intervention to be initiated during work up using validated tools such as the Beck Depression Inventory-II (BDI-II) or Patient Health Questionnaire 9 (PHQ-9) [6, 19, 20]. The BDI-II is a brief validated, easy to use scale which covers all DSM-IV diagnostic criteria for major depressive disorder, and stands as a reliable indicator of symptom severity and suicidal thoughts. BDI-II has high reliability and good correlation with measures of depression and anxiety [21].

The prevalence of pre-transplant depression in our cohort was 20.9%. This is slightly lower than the 26% observed by Okwuosa et al. [12], who examined the effects of depression in 102 post OHT patients. Similar to our findings, no statistically significant differences in survival between depressed and non-depressed patients were identified [12]. Okwuosa and al. concluded that all-cause hospitalizations were significantly higher in the depressed group, and demonstrated no difference in rejection episodes, severity of rejection between groups, or 5 year survival. These observations are in line with our findings.

Pre-existing depression is much more likely to persist into the post-transplant period [6], necessitating aggressive treatment in the pre-transplant period. Zipfel et al. showed severely depressed patients to be at increased risk of mortality after OHT [22]. As seen in our study, depressed patients are more likely to be non-compliant with prescribed medications, which can lead to adverse events in such high risk patient population [23–25]. A variety of published reports provide convincing evidence that occurrences of both compliance – related and psychiatric problems are relatively frequent and often unpredictable. [8, 26, 27]. It is well accepted that depressed patients respond to interventions such as psychotherapy and/or medications post transplantation. Royal et al. assessed the impact of antidepressant use after liver transplantation among patients with documented preoperative depression. Similar to our findings, no differences in survival and organ rejection were observed when comparing treated depressed with non-depressed groups [25].

Infection constitutes one of the more serious complications following OHT. Occurring predominantly within the first year after transplantation [28] infectious complications can account for up to 30% of deaths. This may partially be explained by the use of high dose immunosuppression early after transplantation. With reduction of immunosuppression in the following years, the number of infection related deaths decreases to 10% annually [29]. The reason for an increased number of infections in our depressed cohort is unknown. Paris and al. discovered a statistically significant association between psychiatric problems and risk of infection. Though, a causal association could not be ascribed [30]. In our study, patients with history of depression were more likely to be non-compliant, yet the incidence of rejection was similar between both groups. This may suggest that the immunosuppression regimen played a minor role.

Our study differs from the previous OHT studies in that it sheds light on the importance of pre-transplant evaluation and suggests that depressed patients represent a high-risk cohort that should be identified and managed more aggressively. Additionally, pre-transplant depression, if detected early and treated aggressively (with assistance of a transplant psychiatry/psychology team), does not necessarily portend a poor prognosis. However, despite treatment, these patients remain at a higher risk for morbidity in the post-transplant period and require aggressive 1) monitoring to ensure compliance, and 2) treatment to reduce subsequent hospitalizations.

Our results support the existing body of data suggesting that depression is associated with increased healthcare utilization [31]. It is imperative to identify these patients early. Initiation of appropriate treatment is expected to reduce resource utilization. Despite a relatively small sample size, our findings support a role for early, active intervention in depressed patients.

### Study limitations

The retrospective nature of our study and relatively small sample size may limit the generalizability of these findings. However, all patients underwent standardized psychosocial evaluation performed by a heart transplant social worker prior to transplantation, minimizing potential selection bias. Since the depressed patients were later referred to different consultant psychiatrists, the potential for inter-observer variability must be considered (though, preoperatively all patients were evaluated by the same cardiac behavioral medicine team specializing in advanced heart failure and transplant patients). The retrospective nature further limits this study due to the possibility of the under-diagnosis of depression. It is possible that patients who developed depressive symptoms following OHT were not identified and as a result may have been categorized as non-depressed - further supporting the need for a prospective study that frequently assesses depressive symptoms.

## Conclusion

No survival difference was identified when comparing depressed and non-depressed heart transplant recipients. Though, depressed patients were more likely to have increased number of hospitalizations and infections. A diagnosis of depression should not preclude OHT, but early identification and intervention must be occur. Additional larger prospective studies are needed to validate our findings.

## Abbreviations

BDI-II: Beck Depression Inventory-II
IQR: Inter-quartile range
ISHLT: International Society of Heart and Lung Transplantation
OHT: Orthotopic heart transplantation
PHQ-9: Patient Health Questionnaire 9
UNOS: United Network for Organ Sharing Transplant

## References

[1] Cavanaugh S, Fulanetto LM, Creech SD, Powell LH (2011). Medical illness, past depression and present depression: a predictive triad of in-hospital mortality. Am J Psychiatry.

[2] Hermann C, Brand DS, Kaminsky B, Leibing E, Staats H, Ruger U (1998). Diagnostic groups and depressed mood as predictors of 22-month mortality in medical inpatients. Psychosom Med.

[3] Kuhn WF, Brennan AF, Lacefield PK, Brohm J, Skelton VD, Gray LA (1990). Psychiatric distress during stages of heart transplant protocol. J Heart Transplant.

[4] Lesko LM, Hawkins DR, Akhtar S (1983). Psychological aspects of transplantation medicine. New psychiatric syndromes: DSM-III and beyond.

[5] Mai FM (1993). Psychiatric aspects of heart transplantation. Br J Psychiatry.

[6] Dew MA, Roth LH, Schulberg HC, Simmons RG, Kormos RL, Trzepacz PT, Griffith BP (1996). Prevalence and predictors of depression and anxiety-related disorders during the year after heart transplantation. Gen Hosp Psychiatry.

[7] Zipfel S, Lowe B, Paschke T, Immel B, Lange R, Zimmermann R, Herzog W, Bergmann G (1998). Psychological distress in patients awaiting heart transplantation. J Psychosom Res.

[8] Shapiro PA, Williams DL, Foray AT (1995). Psychosocial evaluation and prediction of compliance problems and morbidity after heart transplantation. Transplantation.

[9] Geller SE, Connolloy T (1997). The influence of psychosocial factors on health transplantation decisions and outcomes. J Transplant Coord.

[10] Maricle RA, Hosenpud JD, Norman DJ (1991). The lack of predictive value of preoperative psychologic distress for postoperative medical outcome in heart transplant recipients. J Heart Lung Transplant.

[11] Skotzko CE, Rudis R, Kobashigawa JA (1999). Psychiatric disorders and outcome following cardiac transplantation. J Heart Lung Transplant.

[12] Okwuosa I, Pumphrey D, Puthumana J, Brown RM, Cotts W (2014). Impact of identification and treatment of depression in heart transplant patients. Cardiovasc Psychiatry Neurol.

[13] Olbrisch ME, Bernedict SM, Ashe K (2002). Psychosocial assessment and care of organ-transplant patients. J Cons Clin Psychol.

[14] Carney RM, Freedland KE (2003). Depression, mortality, and medical morbidity in patients with coronary heart disease. Biol Psychiatry.

[15] Schulz R, Beach SR, Ives DG, Martire LM, Ariyo AA, Kop WJ (2000). Association between depression and mortality in older adults: the cardiovascular health study. Arch Intern Med.

[16] Penninx BWJH, Beekman ATF, Honig A (2001). Depression and cardiac mortality: results from a community-based longitudinal study. Arch Gen Psychiatry.

[17] Barefoot JC, Schroll M (1996). Symptoms of depression, acute myocardial infarction, and total mortality in a community sample. Circulation.

[18] Costanzo MR, Dipchand A, Starling R (2010). The international society of heart and lung transplantation guidelines for the care of heart transplant recipients. J Heart Lung Transplant.

[19] Fusar-Poli P, Picchioni M, Martinelli V (2006). Anti-depressive Therapies After Heart Transplantation. J Heart Lung Transplant.

[20] Wang YP, Gorenstein C (2013). Assessment of depression in medical patients: a systematic review of the utility of the Beck Depression Inventory-II. Clinics (Sao Paulo).

[21] Beck AT, Guth D, Steer RA, Ball R (1997). Screening for major depression disorders in medical inpatients with the Beck Depression Inventory for Primary Care. Behav Res Ther.

[22] Zipfel S, Schneider A, Wild B (2002). Effect of depressive symptoms on survival after heart transplantation. Psychosom Med.

[23] Cukor D, Rosenthal DS, Jindal RM, Brown CD, Kimmel PL (2009). Depression is an important contributor to low medication adherence in hemodialyzed patients and transplant recipients. Kidney Int.

[24] Gehi A, Haas D, Pipkin S, Whooley MA (2005). Depression and medication adherence in outpatients with coronary heart disease: findings from the heart and soul study. Arch Intern Med.

[25] Rogal SS, Landsittel D, Surman O, Chung RT, Rutherford A (2011). Pretransplant depression, antidepressant use, and outcomes of orthotopic liver transplantation. Liver Transpl.

[26] Deal MA, Roth LH, Thompson ME, Kombos RL, Griffith BP (1996). Medical Compliance and its Predictors in the first year after heart transplantation. J Heart Lung Transplant.

[27] Reese RL, Freedland KE, Steinmeyer BC, Rich MW, Rackley JW, Carney RM (2011). Depression and rehospitalization following acute myocardial infarction. Circulation: Cardiovascular Quality and Outcomes.

[28] Wilhelm MJ (2015). Long-term outcome following heart transplantation: current perspective. J Thorac Dis.

[29] Lund LH, Edwards LB, Kucheryavaya AY (2014). The registry of the International Society for Heart and Lung Transplantation: thirty-first official adult heart transplant report--2014; focus theme: Retransplantation. J Heart Lung Transplant.

[30] Paris W, Munchmore J, Pribil A, Zuhdi N, Cooper DKC (1994). Study of the relative incidences of psychosocial factors before and after heart transplantation and the influence of posttransplantation psychosocial factors on heart transplant outcomes. J Heart Lung Transplant.

[31] Lacruz ME, Emeny RT, Haefner S (2012). Relation between depressed mood, somatic comorbidities and health service utilisation in older adults: results from the KORA-Age study. Age Ageing.

